# Autocrine Acetylcholine, Induced by IL-17A via NF*κ*B and ERK1/2 Pathway Activation, Promotes MUC5AC and IL-8 Synthesis in Bronchial Epithelial Cells

**DOI:** 10.1155/2016/9063842

**Published:** 2016-05-19

**Authors:** Angela Marina Montalbano, Giusy Daniela Albano, Anna Bonanno, Loredana Riccobono, Caterina Di Sano, Maria Ferraro, Liboria Siena, Giulia Anzalone, Rosalia Gagliardo, Michael Paul Pieper, Mark Gjomarkaj, Mirella Profita

**Affiliations:** ^1^Institute of Biomedicine and Molecular Immunology “A. Monroy” (IBIM), National Research Council of Italy (CNR), 90146 Palermo, Italy; ^2^Dipartimento di Biomedicina Sperimentale e Neuroscienze Cliniche (BioNec), 90127 Palermo, Italy; ^3^Boehringer Ingelheim Pharma GmbH & Co. KG, 88400 Biberach, Germany

## Abstract

IL-17A is overexpressed in the lung during acute neutrophilic inflammation. Acetylcholine (ACh) increases IL-8 and Muc5AC production in airway epithelial cells. We aimed to characterize the involvement of nonneuronal components of cholinergic system on IL-8 and Muc5AC production in bronchial epithelial cells stimulated with IL-17A. Bronchial epithelial cells were stimulated with recombinant human IL-17A (rhIL-17A) to evaluate the ChAT expression, the ACh binding and production, the IL-8 release, and the Muc5AC production. Furthermore, the effectiveness of PD098,059 (inhibitor of MAPKK activation), Bay11-7082 (inhibitor of IkB*α* phosphorylation), Hemicholinium-3 (HCh-3) (choline uptake blocker), and Tiotropium bromide (Spiriva®) (anticholinergic drug) was tested in our* in vitro* model. We showed that rhIL-17A increased the expression of ChAT, the levels of ACh binding and production, and the IL-8 and Muc5AC production in stimulated bronchial epithelial cells compared with untreated cells. The pretreatment of the cells with PD098,059 and Bay11-7082 decreased the ChAT expression and the ACh production/binding, while HCh-3 and Tiotropium decreased the IL-8 and Muc5AC synthesis in bronchial epithelial cells stimulated with rhIL-17A. IL-17A is involved in the IL-8 and Muc5AC production promoting, via NF*κ*B and ERK1/2 pathway activation, the synthesis of ChAT, and the related activity of autocrine ACh in bronchial epithelial cells.

## 1. Introduction

Epithelial cells can contribute to chronic inflammatory disorders, synthesizing and secreting a variety of proinflammatory cytokines, such as IL-8, which regulates the neutrophil accumulation in the airways of Chronic Obstructive Pulmonary Disease (COPD) subjects [1, 2]. In response to proinflammatory stimuli, IL-8 production is dependent on MAPK and NF*κ*B in epithelial cells [3]. In addition, in human bronchial epithelial cells, the NF*κ*B activation may regulate IL-8 release via ERK/MAP kinase-dependent or kinase-independent processes [4].

Airway epithelial cells contribute to inflammatory process of the airway producing mucins. Muc5AC is the most expressed mucin in the airway epithelium. Cigarette smoke, source of oxidative stress in the airways, is able to induce mucin synthesis in epithelial cells and a subsequent goblet cell production [5]. The mucociliary dysfunction component of COPD is due to mucus hypersecretion coupled with a decrease in mucus transport, and it is an important pathophysiological feature requiring appropriate treatment [6].

IL-17A, a major product of Th17 cells, is implicated in the pathogenesis of several inflammatory and autoimmune diseases [7, 8]. IL-17A is involved in the development and progression of inflammatory diseases of the airways, including allergic asthma, rhinitis, and COPD [9–11]. Recent studies have demonstrated the effect of IL-17A on IL-8 secretion in the airway epithelial cells and in airway smooth muscle cells [9, 12, 13]. Furthermore, IL-17A promotes the growth of airway epithelial cells through ERK-dependent signaling pathway [14] and is able to generate Muc5AC by NF*κ*B in bronchial epithelial cells [15].

Acetylcholine (ACh) is involved in airway inflammation and remodeling processes [16–19]. It is synthesized by Choline Acetyl-Transferase (ChAT) in different cell types (macrophages, T-lymphocytes, fibroblasts, and epithelial cells) acting as an autocrine/paracrine growth factor in regulating various aspects of the innate mucosal defense mechanisms including mucociliary clearance and regulation of macrophage function [20]. Nonneuronal ACh is involved in the activation of bronchial epithelial cells and alveolar macrophages as well as in the release of chemotactic mediators for eosinophils and neutrophils. It contributes to the inflammatory processes of COPD via the activation of muscarinic receptors (MRs) M1, M2, and M3 [15, 21, 22], and it is involved in the Th17 immunity of COPD patients [23]. Cholinergic agonists promote mucociliary clearance [24] and the release of inflammatory mediators from airway epithelial cells [22]. Anticholinergic drugs, currently used in the treatment of COPD, block the activity of muscarinic receptors in the airway secretory cells and in smooth muscle, reducing vagal tone and mucus secretion in COPD [25, 26]. Furthermore,* in vitro*, anticholinergic drugs control the release of LTB4 in the inflammatory cells recovered from induced sputum of COPD [19].

We investigated whether IL-17A, via ERK1/2 and NF*κ*B signal pathway activation, is able to modulate the synthesis of ChAT promoting the* autocrine* ACh release and binding on the cell surface of 16-HBE, in an* in vitro* model of bronchial epithelial cell line. Furthermore, we studied whether the autocrine ACh activity, induced by IL-17A, promotes the production of IL-8 and Muc5AC in bronchial epithelial cells. Finally, we tested the effectiveness of Tiotropium bromide (Spiriva), anticholinergic drug usually used in the treatment of COPD, in our* in vitro* model of bronchial inflammation.

## 2. Materials and Methods

### 2.1. Epithelial Cell Cultures

The SV40 large T antigen-transformed 16-HBE cell line, an immortalized normal bronchial epithelial cell line, or primary normal human bronchial epithelial (N-HBE) cells (ATCC, catalog number PCS-300-010) were used in this study. The source and origin of 16-HBE cells were kindly provided by Dr. D. Gruenert Laboratory (University of California, San Francisco, California) to IBIM-CNR, Italy. The 16-HBE cell line retains the morphology and functions of differentiated bronchial epithelial cells. The cells represent a clonal diploid (2*n* = 6) cell line isolated from human lung. Evidences showed that 16-HBE cells are similar to primary normal human bronchial epithelial (N-HBE) cells and to bronchial epithelial cells from bronchial brushings concerning the response to proinflammatory stimuli and anti-inflammatory drugs [27].

16-HBE cells and N-HBE cells were cultured as adherent monolayers in Eagle's minimum essential medium (MEM) supplemented with 10% heat-inactivated (56°C, 30 min) fetal bovine serum (FBS), 1% MEM (nonessential amino acids, EuroClone), 2 mM L-glutamine, and gentamicin 250 *μ*g/mL at 37°C in a humidified 5% CO_2_ atmosphere.

### 2.2. Stimulation of 16-HBE and N-HBE Cells with Recombinant Human IL-17A

Viable 16-HBE and N-HBE cells (5 × 10^5^) were seeded into six-well plate in MEM 10% FBS and cultured until confluence (70–80%). Cells were then made quiescent by culturing for 24 h in MEM serum-free. The 16-HBE cells were then stimulated with rhIL-17A (0–50 ng/mL) (R&D Systems, Minneapolis, MN, USA) for 30 minutes to test the activation of the intracellular signal pathways and for 24 h to evaluate the intracellular expression of ChAT (protein and mRNA), ACh expression, IL-8 release, and Muc5AC production. The N-HBE cells were stimulated with rhIL-17A 20 ng/mL for 24 h to evaluate the intracellular expression of ChAT (protein and mRNA), ACh expression, IL-8 release, and Muc5AC production.

### 2.3. Stimulation of Bronchial Epithelial Cells with Drugs and Inhibitors

We tested the effects of 2-(2-amino-3-methoxyphenyl)-4*h*-1-benzopyran-4-one (PD098,059, an inhibitor of MAPKK activation, 25 *μ*M) (Sigma Aldrich s.r.l., Milan, Italy) and (*E*)-3-(4-methylphenylsulfonyl)-2-propenenitrile (Bay11-7082, an inhibitor of IkB*α* phosphorylation, 50 *μ*M) (Sigma Aldrich s.r.l., Milan, Italy) in 16-HBE cells stimulated in the presence and absence of rhIL-17A 20 ng/mL. Furthermore, we tested the effect of Hemicholinium-3 (HCh-3) (a potent and selective choline uptake blocker, 50 *μ*M) (Sigma Aldrich, Milan, Italy) in 16-HBE cells stimulated in the presence and absence of rhIL-17A 20 ng/mL. Finally, we studied the effects of anticholinergic compound Tiotropium (Spiriva) (100 nM) (Boehringer Ingelheim Pharma GmbH & Co. KG, Biberach, Germany) in 16-HBE and N-HBE cells stimulated in the presence and absence of rhIL-17A 20 ng/mL. The drugs were added to the cells medium 1% FBS (5% CO_2_ at 37°C) 1 h before the stimulation with rhIL-17A.

### 2.4. Evaluation of ChAT Protein Expression

The expression of ChAT protein was determined in 16-HBE and N-HBE cells treated with rhIL-17A for 24 h or medium alone using indirect label immunofluorescence analyzed by flow cytometry (FACStar Plus analyzer, Becton Dickinson, Mountain View, CA) as previously described [28]. Furthermore, the expression of ChAT protein was measured by western blot analysis as previously described [28]. The primary mouse anti-ChAT antibody (MAB5270, Chemicon, Millipore) was used for both flow cytometry and western blot.

### 2.5. Evaluation of ChAT by Real-Time Quantitative RT-PCR

Total RNA was extracted from 16-HBE cells with TRIzol Reagent (Invitrogen) following the manufacturer's instructions and was reverse-transcribed into cDNA, using M-MLV-RT and oligo(dT)_12–18_ primer (Invitrogen). Quantitative real-time PCR of ChAT transcripts was carried out on StepOnePlus Real-Time PCR System (Applied Biosystems, Foster City, CA, USA) using specific FAM-labeled probe and primers (prevalidated TaqMan Gene Expression Assay for ChAT, Hs00253141 m1, Assays-on-Demand, Applied Biosystems). The expression of ChAT gene was normalized to glyceraldehyde-3-phosphate dehydrogenase (GAPDH) endogenous control gene. Relative quantitation of gene expression was carried out with the comparative Ct method (2^−ΔΔCt^) [29] and it was plotted as fold change compared with untreated cells as the reference sample.

### 2.6. Evaluation of ACh Binding

ACh binding was performed as previously described [30]. The 16-HBE cells were collected, washed in cold PBS, and incubated 1 h at 4°C with a rabbit polyclonal anti-ACh antibody (ab8884, Abcam, Cambridge, UK). This antibody specifically recognized the ACh bound to biological structures such as receptors. After washing with cold PBS, FITC-conjugated polyclonal swine anti-rabbit IgG (Dako, Glostrup, Denmark) was added to the cells for 30 min at 4°C. Fluorescence-positive cells were analyzed using FACSCalibur*™* flow cytometer (Becton Dickinson, Mountain View, CA, USA). The percentage of positive cells was determined from forward scatter (FS) and sideways scatter (SS) patterns. No specific binding as well as background fluorescence was detected by analyzing negative control samples. The results were expressed as fluorescence mean intensity (FMI).

### 2.7. Evaluation of ACh Production

ACh production was performed as previously described [30]. It was measured in protein extracts from cultured 16-HBE cells by a fluorimetric method using a commercial kit (BioVision Research Products, CA, USA, cat. #K615-100). The kit detects choline (Ch) and total choline (TCh) by adding acetylcholine esterase to the reaction that converts ACh into Ch with sensitivity until 50 pmol/well by plotting fluorescence readings (Ex/Em 535/587 nm) against the standard curve. This sensitivity is correspondent to the concentration of 1 *μ*M of TCh or Ch. ACh was evaluated as difference between TCh and Ch. Fluorescence intensity was read using a Wallac 1420 Victor^2^ multilabel counter (PerkinElmer Life Sciences, Turku, Finland). Results were expressed as pmoli/*μ*g protein.

### 2.8. Evaluation of ERK1/2 and NF*κ*B Activation

The activation of ERK1/2 and NF*κ*B pathway was performed by western blot analyses as previously described [30]. We used two rabbit monoclonal antibodies against anti-phospho-ERK1/2 and against anti-phospho-IkB*α* (Cell Signaling Technology, Beverly, MA), respectively, and an anti-*β*-actin antibody (Sigma, St. Louis, MO).

### 2.9. Total and Nuclear/Cytoplasmic Protein Extraction

The cells were washed with cold PBS and nuclear/cytoplasmic extracts were obtained using an NE-PER Nuclear and Cytoplasmic Extraction Kit (Thermo Scientific, Rockford, USA), which ensures efficient cell lysis and extraction of separate cytoplasmic and nuclear protein fractions by centrifugation. Then, 25–30 *μ*g of the lysate was denatured under reducing conditions by boiling for 3 min in 50 mM Tris-HCl (pH 6.8), 1% sodium dodecyl sulfate (SDS), 2%  *β*-mercaptoethanol, and 0.01% bromophenol blue.

### 2.10. Evaluation of the Nuclear Translocation of NF*κ*B-p65

The nuclear and cytoplasmic proteins extracts were separated by SDS-polyacrylamide gel electrophoresis (PAGE) and transferred via electrophoresis onto Immobilon-P membranes (Millipore, Bedford, MA, USA). After transfer, the membranes were blocked overnight at room temperature in PBS containing 3% BSA and 0.5% Tween 20 and then incubated for 1 h at room temperature with anti-NF*κ*B-p65 (C-20) sc-372, Santa Cruz Biotechnology (Santa Cruz, CA, USA). The blot was incubated with the appropriate horseradish peroxidase-conjugated secondary Ab.

### 2.11. Evaluation of Muc5AC by Flow Cytometry

Muc5AC expression was determined using indirect label immunofluorescence. 16-HBE and N-HBE cells were previously fixed with 4% paraformaldehyde in PBS, washed, and permeabilized with PBS (containing 0.1% saponin, 1% sodium azide, and 10% FBS) and incubated with primary antibody, mouse monoclonal anti-Muc5AC clone 45M1 (200 *μ*g/mL, #MS-145-P, NeoMarkers, Fremont, CA). The cells were washed twice and then incubated with FITC-conjugated rabbit polyclonal anti-mouse IgG F(ab′)_2_ (Dako, Glostrup, Denmark) for 30 min at 4°C and then analyzed by flow cytometry analyses (FACSCalibur flow cytometer, Becton Dickinson, Mountain View, CA, USA) supported by CellQuest acquisition and data analysis software (Becton Dickinson, Mountain View, CA, USA). The cells were considered Muc5AC positive when their fluorescence 1 (FL1) was greater than a gate set to exclude all cells in the FL1 peak from an isotype matched control antibody [31].

### 2.12. Evaluation of Muc5AC by Western Blot Analysis

16-HBE and N-HBE cells were lysed with lysis buffer (10 mM Tris-HCl (pH 7.4), 50 mM NaCl, 5 mM EDTA, and 1% NP-40 NONIDET) containing protease and phosphatases inhibitors and frozen at −20°C. After being defrozen, samples were centrifuged at 12000 rpm for 20 min, supernatants were recovered, and the protein concentration was quantified by Bradford solution. Equal amounts of cell extract proteins (40 *μ*g/lane) were separated via SDS-PAGE electrophoresis in 8% acrylamide-bisacrylamide (80 : 1) under nonreducing conditions [32, 33]. The resulting gel was equilibrated in the transfer buffer. The proteins were then transferred electrophoretically to nitrocellulose membranes that were incubated with 5% fat-free skimmed milk in PBS for 1 h and then incubated with the same antibody and then western blot (dilution 1 : 500, overnight at 4°C). Subsequently, the membrane was washed and incubated for 1 h with a secondary antibody conjugated to HRP. After wash in PBS 1x, detection was performed with an enhanced chemiluminescence system (Ambion, Austin, TX) followed by autoradiography.

### 2.13. Measurement of IL-8 Production

The levels of IL-8 were determined in 16-HBE supernatants using a commercial ELISA kit (R&D Systems, Inc., MN, USA), according to the manufactures' specifications. The lower detection limit for IL-8 was <5 pg/mL. Furthermore, the levels of IL-8 expression were determined in 16-HBE and N-HBE protein extracts by western blot using a mouse monoclonal anti-IL-8 (dilution 1 : 100, overnight at 4°C) (sc-8427, Santa Cruz Biotechnology, Inc.) and a sheep anti-mouse IgG-HRP (dilution 1 : 1000) (NA931, GE Healthcare UK, Little Chalfont, Buckinghamshire, UK) as primary and secondary antibody, respectively.

### 2.14. RNA Interference of ChAT Protein Synthesis

Cell transfection was performed according to the manufacturer's instructions (72 h at 37°C) in siPORT NeoFX Transfection Agent (Ambion, Inc., Texas, USA) diluted in Opti-MEM (Gibco Invitrogen, Milan, Italy) and ChAT siRNA (20 *μ*M, s-2969 Ambion, Inc., Texas, USA) diluted in Opti-MEM for a final concentration of 30 nM. A siRNA (20 *μ*M, Ambion, Inc., Texas, USA), containing a scrambled sequence without significant homology to the human genome, was used as negative control. Knockdown efficiency was assessed on ChAT protein expression by western blot analysis. Furthermore, the effect of ChAT siRNA was checked in IL-8 measurement and in Muc5AC production obtained as mentioned above.

### 2.15. Gel Images Evaluation

Gel images were taken with EPSON GT-6000 scanner and then were imported into National Institutes of Health Image analysis 1.61 program to determine band intensities. Data are expressed as arbitrary densitometric units (ADU) corrected against the density of *β*-actin bands.

### 2.16. Statistical Analysis

We tested normal distribution of the data with the* Kolmogorov-Smirnov test*. Analysis of variance (ANOVA) corrected with Fisher's test and *t*-test was used for comparisons. Data were expressed as mean ± standard deviation (SD). A *p* value < 0.05 was considered statistically significant.

## 3. Results

### 3.1. rhIL-17A Increased ChAT Protein Expression and ACh Binding and Production in 16-HBE Cells

**Figure 1:**
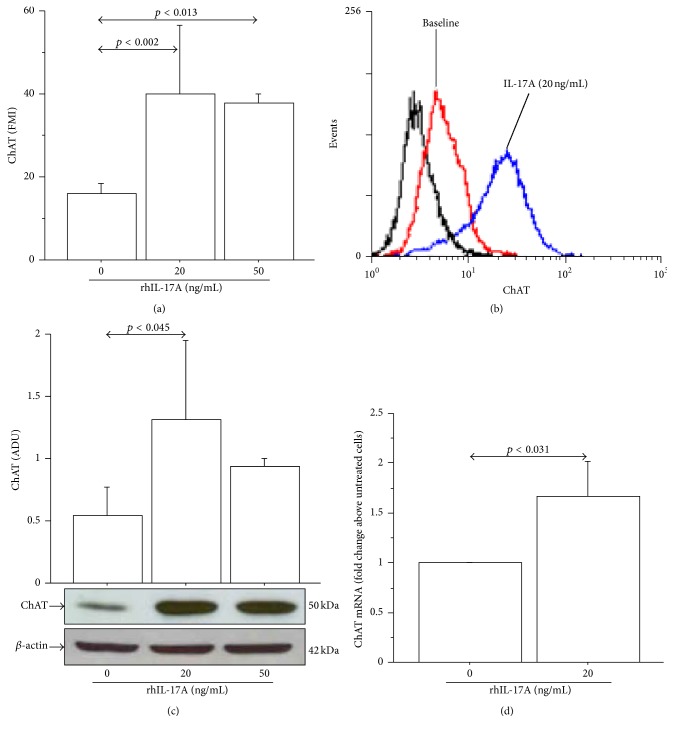
rhIL-17A increased ChAT protein expression and mRNA in 16-HBE cells. Cells were stimulated with rhIL-17A (0–50 ng/mL) for 24 h to evaluate ChAT protein expression (a) by flow cytometry. Bars represent mean ± SD of fluorescence mean intensity (FMI) of three separate experiments. Representative (b) flow cytometry analysis and western blot (c) are shown. Bars represent mean ± SD of arbitrary densitometric units (ADU). Representative western blot analysis of ChAT protein and *β*-actin is shown. (d) Cells were stimulated with rhIL-17A (0–20 ng/mL) for 24 h to evaluate ChAT mRNA levels by RT-PCR. Bars represent mean ± SD of arbitrary units of three separate experiments and were plotted as fold change compared to untreated cells. Statistical analysis was performed by ANOVA test followed by Fisher's PLSD multiple comparison test or Student's *t*-test. The black curve represents the anti-IgG isotype negative control antibody.

**Figure 2:**
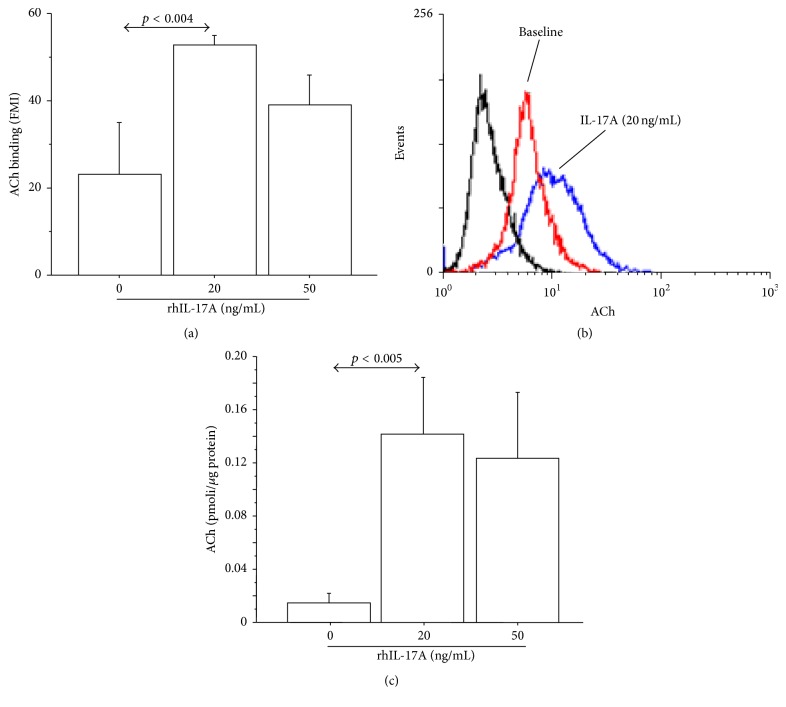
rhIL-17A increased endogenous ACh binding and production in 16-HBE cells. Cells were stimulated with rhIL-17A (0–50 ng/mL) for 24 h to evaluate (a) ACh binding by flow cytometry. Bars represent mean ± SD of fluorescence mean intensity (FMI) of three separate experiments. (b) Representative flow cytometry analysis is shown. (c) ACh production expressed as pmoli/*μ*g protein. Bars represent mean ± SD of three different experiments. Statistical analysis was performed by ANOVA test followed by Fisher's PLSD multiple comparison test. The black curve represents the anti-IgG isotype negative control antibody.

The stimulation of 16-HBE cells with rhIL-17A (20 and 50 ng/mL for 24 h) significantly increased ChAT protein expression compared with unstimulated cells by both flow cytometry (Figures 1(a) and 1(b)) and western blot analyses (Figure 1(c)). The stimulation of the cells with rhIL-17A 20 ng/mL reached the higher levels of ChAT synthesis (Figure 1). Accordingly, we showed that the levels of ChAT mRNA, obtained by RT-PCR, significantly increased in 16-HBE cells stimulated with rhIL-17A 20 ng/mL compared with unstimulated cells (Figure 1(d)). Finally, we showed that rhIL-17A (20 and 50 ng/mL for 24 h) significantly increased the ACh binding (Figures 2(a) and 2(b)) and production (Figure 2(c)) compared with unstimulated cells. The stimulation of the cells with rhIL-17A 20 ng/mL reached the higher levels of ACh binding and production (Figure 2).

### 3.2. NF*κ*B and ERK1/2 Pathway Activation

**Figure 3:**
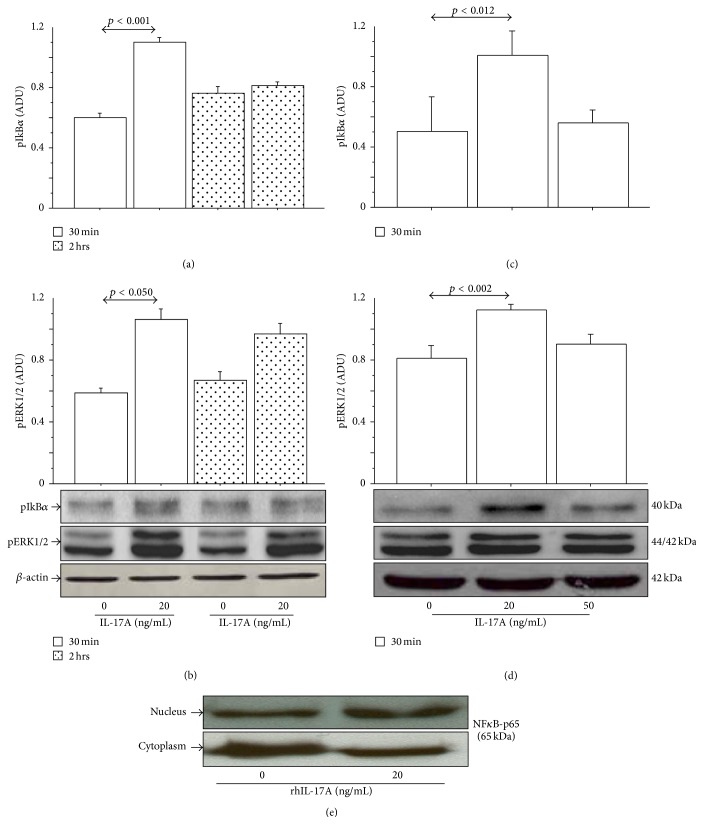
rhIL-17A increased pIkB*α* and pERK1/2 in 16-HBE cells. Cells were stimulated with rhIL-17A (0–20 ng/mL) for 30 min and 2 h to evaluate (a) pIkB*α* and (b) pERK1/2 by western blot. Cells were stimulated with rhIL-17A (0–50 ng/mL) for 30 min to evaluate (c) pIkB*α* and (d) pERK1/2 by western blot. Bars represent the mean ± SD of arbitrary densitometric units (ADU) of three separate experiments. Representative western blot analyses of pIkB*α*, pERK1/2 protein, and *β*-actin are shown. (e) NF*κ*B-p65 subunit in nuclear and cytosol extracts from 16-HBE cells stimulated with rhIL-17A 20 ng/mL. Western blot analysis is shown. Statistical analysis was performed by Student's *t*-test and by ANOVA test followed by Fisher's PLSD multiple comparison test.

The pIkB*α* and pERK1/2 were significantly increased in 16-HBE cells stimulated with rhIL-17A 20 ng/mL for 30 min in comparison with untreated cells, reaching higher levels when compared with the cells stimulated for 2 h with rhIL-17A 20 ng/mL (Figures 3(a) and 3(b)). The dose-response curve showed a significant increase of pIkB*α* and pERK1/2 in 16-HBE cells stimulated with rhIL-17A 20 ng/mL in comparison with unstimulated cells. The levels of pIkB*α* and pERK1/2 shaped in 16-HBE cells stimulated with rhIL-17A 50 ng/mL in comparison with the cells stimulated with rhIL-17A 20 ng/mL are shown in Figures 3(c) and 3(d). Finally, we showed an increase of NF*κ*B-p65 nuclear translocation in 16-HBE cells stimulated with rhIL-17A 20 ng/mL compared with untreated cells (Figure 3(e)).

### 3.3. Effect of ERK1/2 and IkB*α* Inhibitors

**Figure 4:**
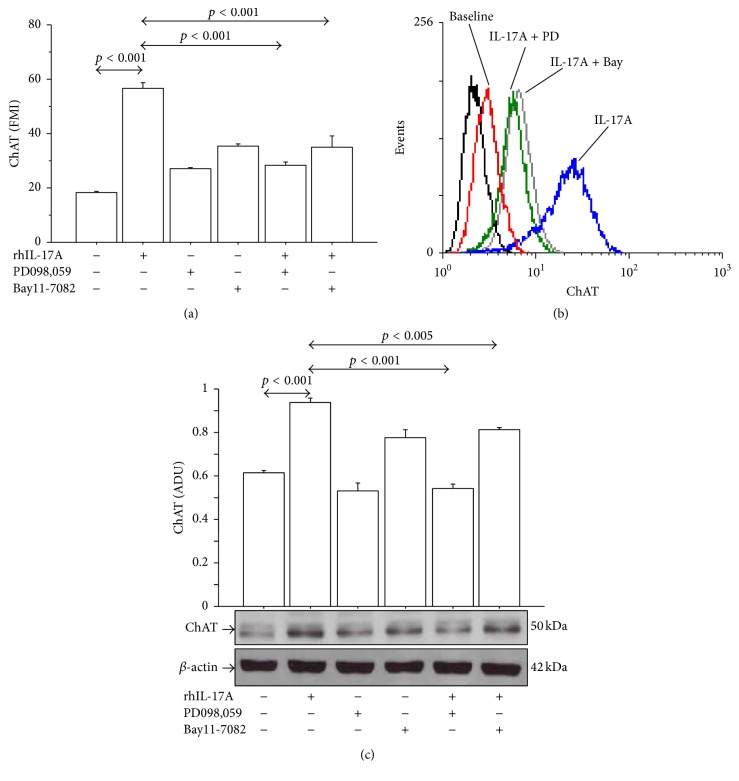
Effects of PD098,059 (25 *μ*M) and Bay11-7082 (50 *μ*M) on ChAT protein expression in 16-HBE cells. Cells were preincubated for 1 h with PD098,059 (25 *μ*M) or Bay11-7082 (50 *μ*M) and then stimulated with rhIL-17A 20 ng/mL for 24 h to evaluate ChAT protein expression (a) by flow cytometry. Bars represent mean ± SD of fluorescence mean intensity (FMI) of three separate experiments. Representative (b) flow cytometry analysis and western blot (c) are shown. Bars represent mean ± SD of arbitrary densitometric units (ADU). Representative western blot is shown. Statistical analysis was performed by ANOVA test followed by Fisher's PLSD multiple comparison test. The black curve represents the anti-IgG isotype negative control antibody.

**Figure 5:**
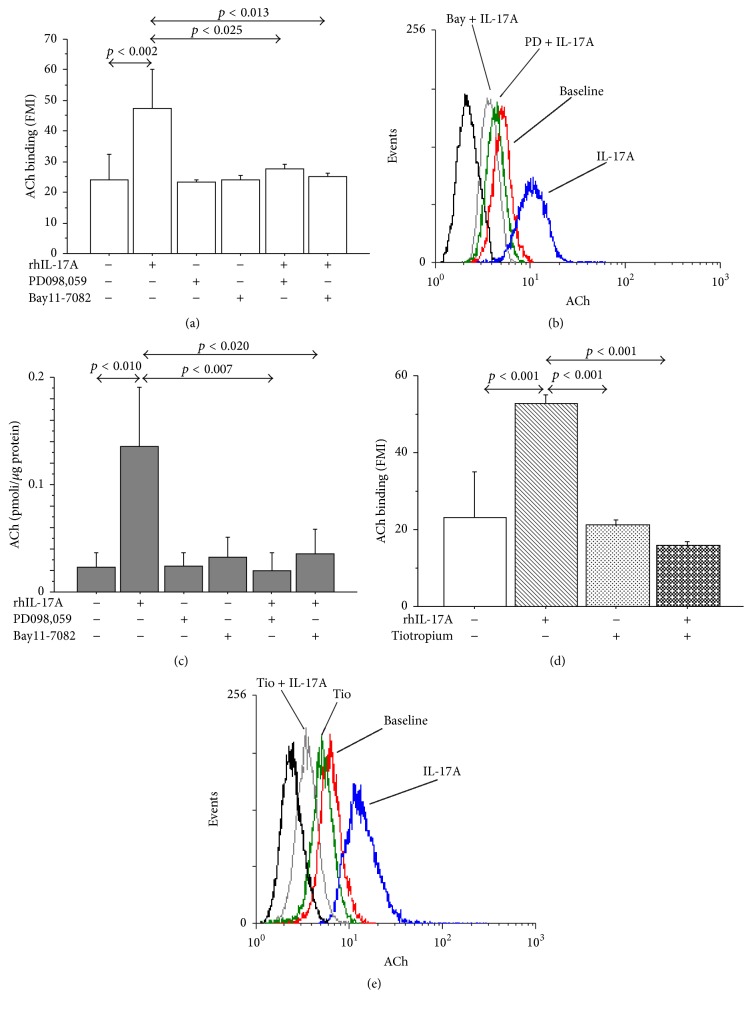
Effects of PD098,059 (25 *μ*M), Bay11-7082 (50 *μ*M), and Tiotropium (100 nM) on ACh in 16-HBE cells. Cells were preincubated for 1 h with PD098,059 (25 *μ*M) or Bay11-7082 (50 *μ*M) and then stimulated with rhIL-17A 20 ng/mL for 24 h to evaluate (a) ACh binding by flow cytometry. Bars represent mean ± SD of fluorescence mean intensity (FMI). (b) Representative flow cytometry is shown. (c) ACh production, expressed as pmoli/*μ*g protein. Bars represent mean ± SD of three different experiments. Then, 16-HBE cells were preincubated for 1 h with Tiotropium (100 nM) and then stimulated with rhIL-17A 20 ng/mL for 24 h to evaluate (d) ACh binding by flow cytometry. Bars represent mean ± SD of fluorescence mean intensity (FMI). (e) Representative flow cytometry is shown. Statistical analysis was performed by ANOVA test followed by Fisher's PLSD multiple comparison test. The black curve represents the anti-IgG isotype negative control antibody.

The preincubation of 16-HBE cells with PD098,059 or Bay11-7082 significantly reduced the expression of ChAT protein in 16-HBE cells stimulated with rhIL-17A 20 ng/mL for 24 h by both flow cytometry (Figures 4(a) and 4(b)) and western blot analysis (Figure 4(c)). Accordingly, the pretreatment of the cells with PD098,059 and Bay11-7082 significantly decreased ACh binding (Figures 5(a) and 5(b)) and production (Figure 5(c)) in 16-HBE cells stimulated with rhIL-17A 20 ng/mL compared with the cells stimulated with rhIL-17A alone. Finally, the use of Tiotropium significantly decreased the ACh binding in 16-HBE cells stimulated with rhIL-17A 20 ng/mL compared with the cells stimulated with rhIL-17A alone (Figures 5(d) and 5(e)).

### 3.4. Effect of HCh-3 and Tiotropium on IL-8 Production and Muc5AC Expression

**Figure 6:**
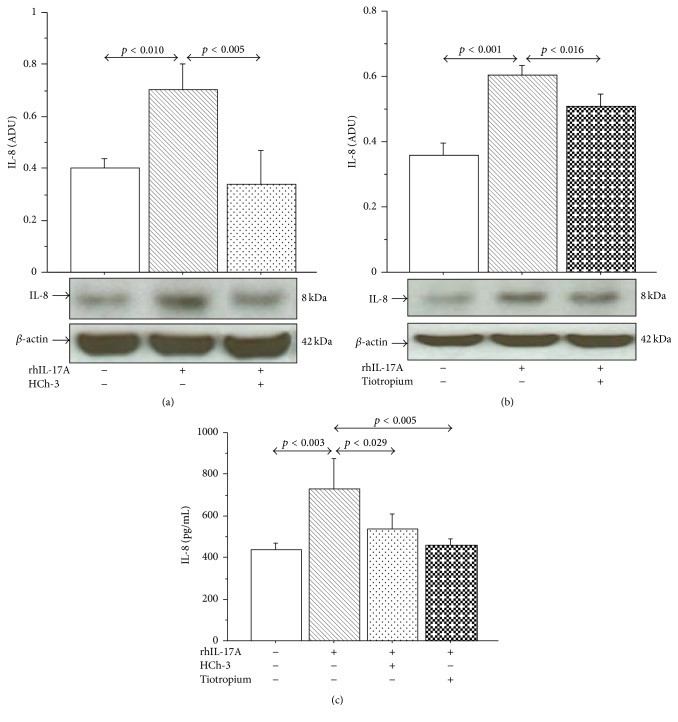
Effect of Hemicholinium-3 (HCh-3) or Tiotropium on IL-8 production in 16-HBE cells. Cells were preincubated for 1 h with HCh-3 (50 *μ*M) or Tiotropium (100 nM) and then stimulated with rhIL-17A 20 ng/mL for 24 h to evaluate ((a)-(b)) IL-8 expression by western blot. Bars represent mean ± SD of arbitrary densitometric units (ADU). Representative western blot analyses of IL-8 protein and *β*-actin are shown. (c) IL-8 release (pg/mL, by ELISA). The values shown are the mean ± SD of three separate experiments. Statistical analysis was performed by ANOVA test followed by Fisher's PLSD multiple comparison test.

**Figure 7:**
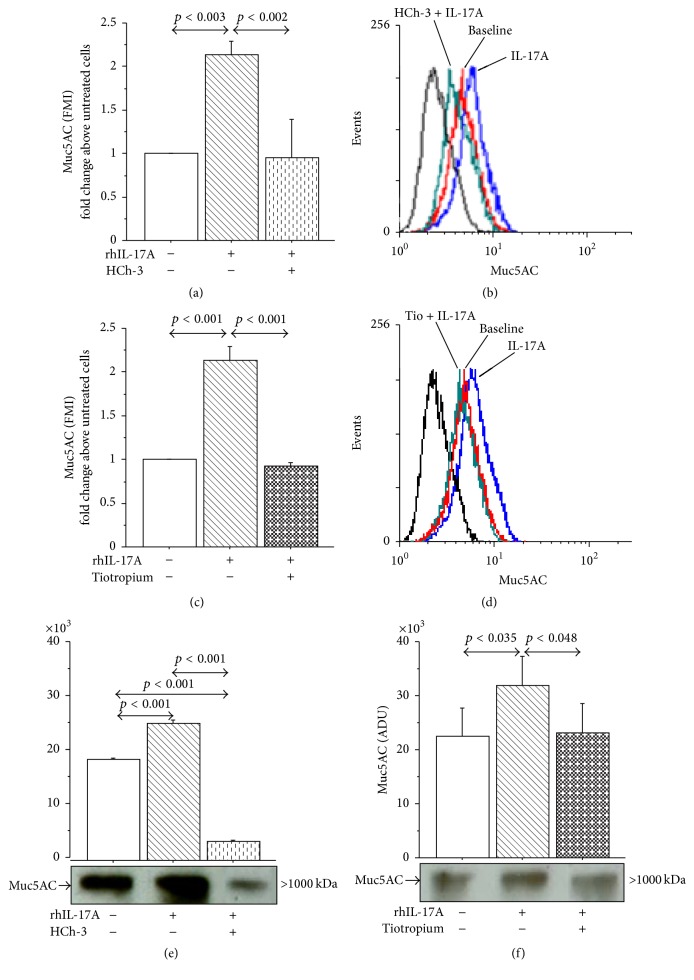
Effect of Hemicholinium-3 (HCh-3) or Tiotropium on Muc5AC production in 16-HBE cells. Cells were incubated for 1 h with HCh-3 (50 *μ*M) or Tiotropium (100 nM) before addition of rhIL-17A 20 ng/mL for 24 h to evaluate ((a)–(d)) Muc5AC protein expression by flow cytometry. Bars represent mean ± SD of fluorescence mean intensity (FMI) of three separate experiments. Data were plotted as fold change compared to untreated cells. Representative flow cytometry of Muc5AC is shown. ((e)-(f)) Muc5AC protein expression by western blot. Bars represent mean ± SD of arbitrary densitometric units (ADU) of three different experiments. Representative western blot analysis of Muc5AC protein is shown. Statistical analysis was performed by ANOVA followed by Fisher's PLSD multiple comparison test. The black curve represents the anti-IgG isotype negative control antibody.

The stimulation of 16-HBE cells with rhIL-17A 20 ng/mL for 24 h significantly increased the levels of IL-8 protein expression and release compared to unstimulated cells (Figure 6). The pretreatment of the cells with HCh-3 (50 *μ*M) or Tiotropium (100 nM) significantly reduced the levels of IL-8 protein expression (Figures 6(a) and 6(b)) and release (Figure 6(c)) in 16-HBE cells stimulated with rhIL-17A 20 ng/mL compared with the cells stimulated with rhIL-17A alone. Additionally, the stimulation of 16-HBE cells with rhIL-17A 20 ng/mL for 24 h significantly increased Muc5AC expression, compared with unstimulated cells evaluated by both flow cytometry and western blot analysis (Figure 7). The pretreatment of the cells with HCh-3 (50 *μ*M) or Tiotropium (100 nM) significantly reduced the Muc5AC protein expression in 16-HBE cells stimulated with rhIL-17A 20 ng/mL compared with the cells stimulated with rhIL-17A alone (Figure 7).

### 3.5. Effect of ChAT Silencing on Muc5AC and IL-8 Production in 16-HBE Cells

**Figure 8:**
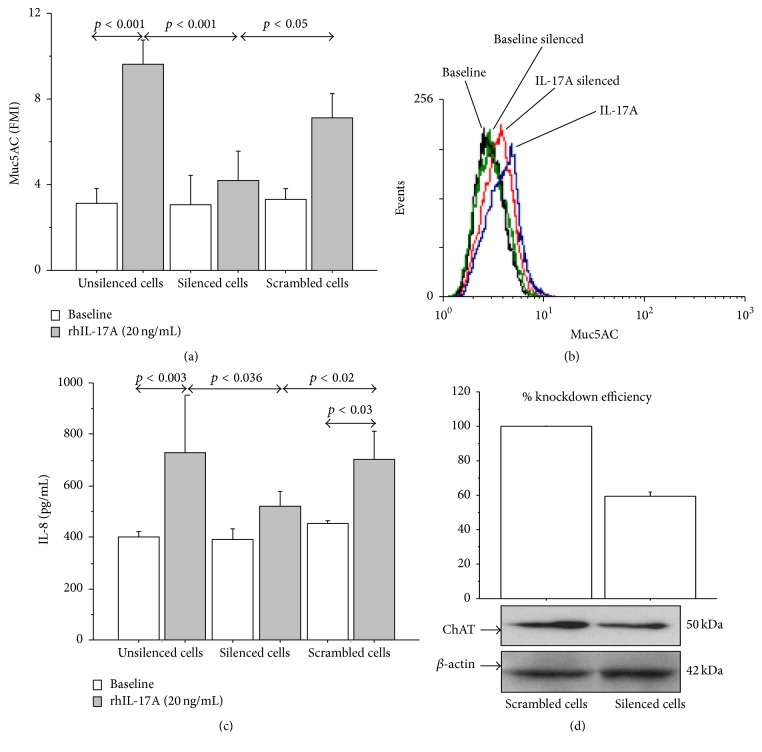
Silencing of ChAT mRNA reduced Muc5AC expression and IL-8 release in 16-HBE cells stimulated with rhIL-17A 20 ng/mL for 24 h. Cells were stimulated and analyzed to evaluate (a) Muc5AC expression in unsilenced cells and in cells transfected with siRNA for ChAT or scrambled siRNA by flow cytometry. Bars represent mean ± SD of fluorescence mean intensity (FMI) of three separate experiments. (b) Representative flow cytometry of Muc5AC is shown. (c) IL-8 release (pg/mL, by ELISA) in unsilenced cells and in cells transfected with siRNA for ChAT or scrambled siRNA. The values shown are the mean ± SD for three separated experiments. (d) Knockdown efficiency (%) of ChAT mRNA on ChAT protein expression. Bars represent mean ± SD of three separate experiments. Statistical analysis was performed by ANOVA test followed by Fisher's PLSD multiple comparison test.

The temporary transfection of 16-HBE cells with ChAT siRNA generated a significant decrease in Muc5AC expression (Figures 8(a) and 8(b)) and IL-8 release (Figure 8(c)) in 16-HBE cells stimulated with rhIL-17A 20 ng/mL, when compared with unsilenced or scrambled cells stimulated with rhIL-17A. Knockdown efficiency of ChAT mRNA (40 ± 2.5%) was obtained comparing the levels of ChAT protein expression in silenced and scrambled unstimulated 16-HBE cells (Figure 8(d)).

### 3.6. Effect of IL-17A on ChAT, Muc5AC, and IL-8 Production in Primary N-HBE Cells

**Figure 9:**
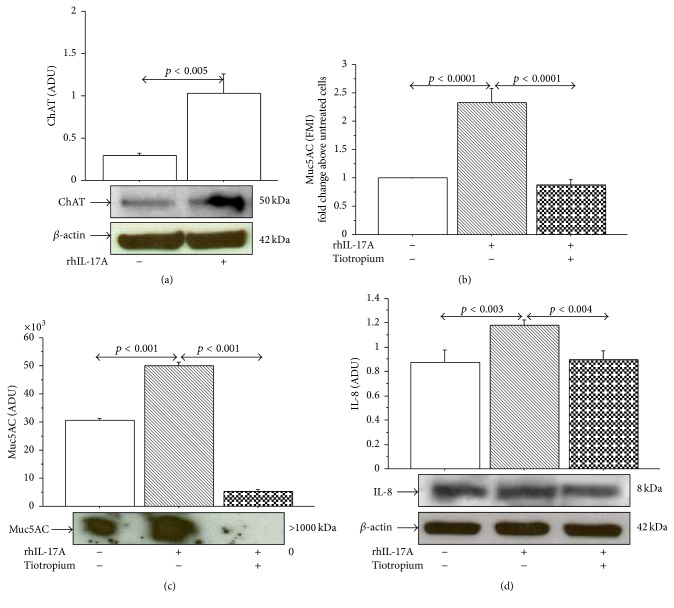
Effect of Tiotropium on ChAT, Muc5AC, and IL-8 in N-HBE cells. Cells were incubated for 1 h with Tiotropium (100 nM) before addition of rhIL-17A 20 ng/mL for 24 h to evaluate (a) ChAT protein expression by western blot. Bars represent mean ± SD of arbitrary densitometric units (ADU). Representative western blot analysis of ChAT protein and *β*-actin is shown. (b) Muc5AC protein expression by flow cytometry. Bars represent mean ± SD of fluorescence mean intensity (FMI) of three separate experiments and were plotted as fold change compared to untreated cells. (c) Muc5AC protein expression by western blot. Bars represent mean ± SD of arbitrary densitometric units (ADU) of three different experiments. Representative western blot analysis of Muc5AC protein is shown. (d) IL-8 expression by western blot. Bars represent mean ± SD of arbitrary densitometric units (ADU). Representative western blot analyses of IL-8 protein and *β*-actin are shown. Statistical analysis was performed by ANOVA followed by Fisher's PLSD multiple comparison test. *p* < 0.05 was accepted as statistically significant.

The stimulation of N-HBE cells with rhIL-17A 20 ng/mL for 24 h significantly increased the levels of ChAT, Muc5AC, and IL-8 protein expression compared with unstimulated cells. The pretreatment with Tiotropium (100 nM) significantly reduced the levels of Muc5AC and IL-8 protein expression in N-HBE cells stimulated with rhIL-17A 20 ng/mL (Figure 9).

## 4. Discussion

In our* in vitro *model, we demonstrated for the first time that IL-17A, via ERK1/2 and NF*κ*B pathway activation, is able to generate an increased synthesis of ChAT protein promoting ACh production and binding in human bronchial epithelial cells. The autocrine ACh activity observed in the presence of IL-17A increased the levels of IL-8 and Muc5AC production in human bronchial epithelial cells. Finally, anticholinergic drugs such as Tiotropium (Spiriva) might have anti-inflammatory and antisecretory properties by the control of ACh activity generated in bronchial epithelial cells stimulated with IL-17A.

Much of our knowledge comes from the interactions between environmental and inflammatory stimuli, and the airway epithelium has been derived extensively from* in vitro* cell culture models using transformed 16-HBE cells, representing an invaluable model in understanding the physiological properties of human airway epithelium [22, 27, 34]. In accordance with this concept, our study is based principally on the use of an immortalized airway epithelial cell line, 16-HBE. Nevertheless, to strengthen the message of our findings we performed some experiments using commercially available primary bronchial epithelial cells.

ACh, classically known as a parasympathetic neurotransmitter, is able to affect the inflammatory processes in COPD [19, 22, 35]. Th17 cells producing IL-17A are associated with nonneuronal ACh in the systemic inflammation of COPD patients [23]. The stimulation of cholinergic receptors enhances IL-10 and IL-17A and inhibits IFN-*γ* secretion in murine spleen T-cells suggesting a link between T-cell, cholinergic receptors, and Th17 lineages [36]. Furthermore, it was observed that IL-17A is a proinflammatory cytokine involved in modulating airway immune response in several aspects covering both the innate and the adaptive immunity in chronic lung disorders [37–39]. Bronchial epithelial cells from COPD patients as well as 16-HBE cell line express IL-17 receptor [40]. In light of this background, we hypothesized a link between IL-17A and the production of autocrine growth factor ACh in bronchial epithelial cells. Accordingly, we found that rhIL-17A is able to increase the basal levels of ChAT protein (the enzyme involved in the synthesis of ACh) synthesis, the levels of autocrine ACh, and the related binding to cellular surface of 16-HBE cells. RhIL-17A affects the synthesis of ChAT protein also in our* in vitro* experiments with primary epithelial cells from normal donors (N-HBE). These findings might suggest that Th17 immunity is involved in the ChAT/ACh activity promoting the bronchial epithelial cells activation during inflammatory process of the airways.

A relevant number of studies identified that IL-17A promotes the activation of bronchial epithelial cells through ERK1/2 or NF*κ*B dependent signaling pathway [14, 15]. The activation of MEK/ERK cascade regulates ChAT promoter via NF*κ*B signal pathway in transfected CHP126 neuroepithelioma cells [41]. Accordingly, we observed higher levels of ERK1/2 and IkB*α* phosphorylation in 16-HBE cells stimulated with rhIL-17A 20 ng/mL, suggesting the involvement of MEK/ERK and NF*κ*B pathway activation in our* in vitro* model. Furthermore, looking at NF*κ*B-p65 in nuclear extract (as upstream of pIkB), we underlined an increase of the nuclear translocation of NF*κ*B in 16-HBE cells stimulated with IL-17A 20 ng/mL. These findings might provide evidences for the involvement of NF*κ*B pathway in bronchial epithelial cells stimulated with IL-17A. To demonstrate that rhIL-17A regulates ChAT synthesis via MEK/ERK cascade and via NF*κ*B pathway, we preincubated with PD098,059 (inhibitor of MAPKK activation) and Bay11-7082 (inhibitor of IkB*α* phosphorylation) the 16-HBE cells stimulated with rhIL-17A. The use of the specific inhibitors reduced the levels of ChAT protein synthesis and ACh production and binding in the cells stimulated with rhIL-17A suggesting the involvement of MEK/ERK cascade and via NF*κ*B pathway in this mechanism of synthesis. However, we observed the dose-response curve of rhIL-17A peaks at 20 ng/mL for ERK1/2 and IkB*α* signaling to shape at the concentration of IL-17A 50 ng/mL. In contrast to these data, we showed persistence of ChAT synthesis and ACh production and binding. These findings might suggest that the higher concentration of rhIL-17A might be involved in the ChAT synthesis, independently of the MEK/ERK cascade and NF*κ*B pathway, by the activation of other intracellular signal pathways. However, in this study we focused our attention on the effect of rhIL-17A 20 ng/mL. Nevertheless, we suggest that further studies will be necessary to better clarify the role of intracellular signal pathway involved in ChAT synthesis and autocrine ACh activity of bronchial epithelial cells stimulated with rhIL-17A 50 ng/mL.

Th17 immunity has an emerging role in the induction of neutrophilic airway inflammation [9, 12, 13] and Muc5AC production [15]. The reduced sensitivity to GCs related to Th17 immunity has been clinically associated with neutrophilic airway inflammation [42, 43], but it is still largely unclear which cellular and molecular mechanisms contribute to this phenomenon. IL-17A activated the p38, extracellular signal-related kinase (ERK), and phosphoinositide-3-kinase (PI3K) pathways inducing GC insensitivity [34]. Furthermore, mechanisms whereby pharmacological agents such as glucocorticoids repress mucin gene expression are not well studied, although they might be important for formulating therapeutic interventions in chronic lung diseases [44]. Preclinical studies have demonstrated that stimulation with cholinergic agonists (ACh, methacholine) or cholinergic antagonists (atropine), targeting muscarinic receptors, increases mucociliary clearance [24, 45, 46]. Accordingly, anticholinergic drugs block muscarinic receptors activity on airway secretory cells and smooth muscle and so, theoretically, may reduce vagal tone and mucus secretion in COPD facilitating cough-induced mucus clearance [25, 26]. Consistent with this suggestion is the observation that Tiotropium reduces levels of mucus secretion in patients with COPD [47]. Furthermore, Tiotropium reduces exacerbation frequency in COPD, but this effect does not appear to be due to a reduction in IL-8 systemic inflammation [48] and is able to control IL-8 release in epithelial cells* in vitro *[22, 49]. In this scenario, we speculate that the blockade of IL-17A downstream, regarding ChAT synthesis, and autocrine ACh production might represent a new strategy for therapeutic intervention in GC insensitivity of airway inflammation using anticholinergic drugs. Accordingly, we showed that the use of Tiotropium is able to reduce autocrine ACh binding. These results suggest the involvement of mAChRs in the activation of bronchial epithelial cells stimulated with IL-17A. Furthermore, we observed that the use of muscarinic receptor antagonists such as Tiotropium is able to control the Muc5AC and IL-8 production blocking the activity of ACh bound to mAChRs generated by IL-17A in bronchial epithelial cells. We support this observation showing the effect of HCh-3 (choline uptake blocker) that, blocking the synthesis of autocrine ACh, reduced the Muc5AC and IL-8 production in 16-HBE cells stimulated with rhIL-17A. Finally, we observed that the ChAT enzyme RNA interference promoted the reduction of Muc5AC and IL-8 production in the 16-HBE cells stimulated with rhIL-17A rather than in both unsilenced and scrambled cells stimulated with rhIL-17A. These results might suggest the potential involvement of ChAT/ACh pathway in the regulation of mucin production and inflammation in bronchial epithelial cells during chronic airway disease. However, rhIL-17A did not affect the expression of mAChRs (M1–3) in 16-HBE cells (data not shown). In this scenario, our* in vitro* model suggests the potential relevant contribution of anticholinergic drugs to the blockade of mucus and inflammatory secretion during COPD exacerbations. Accordingly, we showed that Tiotropium reduced MUC5AC and IL-8 production in the experiment performed with 16-HBE and primary N-HBE cells stimulated with rhIL-17A. Nevertheless, further* “in vivo” *clinical study should be necessary to clarify the role of anticholinergic drugs in the control of mucus and inflammatory mediator secretion from bronchial epithelial cells during IL-17A mediated airway inflammation.

## 5. Conclusions

**Figure 10:**
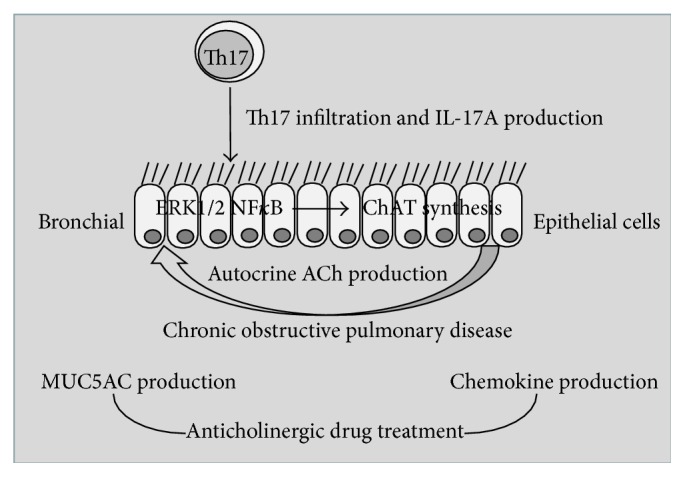
Autocrine acetylcholine induced by IL-17A, via NF*κ*B and ERK1/2 pathway activation, promotes MUC5AC and IL-8 synthesis in bronchial epithelial cells. Effect of anticholinergic drugs.

To our knowledge, this is the first descriptive study demonstrating the involvement of IL-17A in the induction of the ChAT/ACh activity in bronchial epithelial cells promoting inflammation and mucus secretion in the airways. The ability of muscarinic receptor antagonists, including Tiotropium, to modulate IL-17A activity might open up perspectives for novel therapeutic strategies in the control of Th17 immunity during the airway inflammation and the mucus secretion of COPD patients (Figure 10).

## Abbreviations

COPD: Chronic Obstructive Pulmonary Disease
IL-17A: Interleukin-17A
ChAT: Choline Acetyl-Transferase
ACh: Acetylcholine
MRs: Muscarinic receptors
Muc5AC: Mucin 5 AC
IL-8: Interleukin-8
PD098,059: 2-(2-Amino-3-methoxyphenyl)-4*h*-1-benzopyran-4-one, C_16_H_13_NO_3_
Bay11-7082: (*E*)-3-(4-Methylphenylsulfonyl)-2-propenenitrile, C_10_H_9_NO_2_C
HCh-3: Hemicholinium-3, C_24_H_34_Br_2_N_2_O_4_
Tiotropium, Spiriva: (1*α*, 2*β*, 4*β*, 5*α*, 7*β*)-7-[(Hydroxydi-2-thienylacetyl)oxy]-9,9-dimethyl-3-oxa-9-azoniatricyclo[3.3.1.02,4], C19H22NO4S2Br_H2O.
